# The crystal structure of the triclinic polymorph of 1,4-bis­([2,2′:6′,2′′-terpyridin]-4′-yl)benzene

**DOI:** 10.1107/S2056989019015810

**Published:** 2019-11-29

**Authors:** Alexander E. Sedykh, Dirk G. Kurth, Klaus Müller-Buschbaum

**Affiliations:** a Institute of Inorganic and Analytical Chemistry, Justus-Liebig-Universität Giessen, Heinrich-Buff-Ring 17, 35392 Giessen, Germany; b Institute of Inorganic Chemistry, Julius-Maximilians-University Wüzburg, Am Hubland, 97074 Würzburg, Germany; c Lehrstuhl für Chemische Technologie der Materialsynthese, Julius-Maximilians-University Würzburg, Röntgenring 11, 97070 Würzburg, Germany

**Keywords:** crystal structure, terpyridine, C—H⋯π inter­actions, offset π–π inter­actions

## Abstract

The title triclinic polymorph (Form I) of 1,4-bis­([2,2′:6′,2′′-terpyridin]-4′-yl)benzene, C_36_H_24_N_6_, was formed in the presence of the Lewis acid yttrium trichloride in an attempt to obtain a coordination compound. The crystal structure of the ortho­rhom­bic polymorph (Form II), has been described previously [Fernandes *et al.* (2010 ▸). *Acta Cryst.* E**66**, o3241–o3242]. The asymmetric unit of Form I consists of half a mol­ecule, the whole mol­ecule being generated by inversion symmetry with the central benzene ring being located about a crystallographic centre of symmetry. The side pyridine rings of the 2,2′:6′,2′′-terpyridine (terpy) unit are rotated slightly with respect to the central pyridine ring, with dihedral angles of 8.91 (8) and 10.41 (8)°. Opposite central pyridine rings are coplanar by symmetry, and the angle between them and the central benzene ring is 49.98 (8)°. The N atoms of the pyridine rings inside the terpy entities, N⋯N⋯N, lie in *trans–trans* positions. In the crystal, mol­ecules are linked by C—H⋯π and offset π–π inter­actions [inter­centroid distances are 3.6421 (16) and 3.7813 (16) Å], forming a three-dimensional structure.

## Abstract 2

A triclinic polymorph of 1,4-bis­([2,2′:6′,2′′-terpyridin]-4′-yl)benzene was obtained under solvothermal conditions.

## Chemical context   

1,4-Di([2,2′:6′,2′′-terpyridin]-4′-yl)benzene has been used as a ligand in the formation of mononuclear complexes (Santoni *et al.*, 2013 ▸; Laramée-Milette & Hanan, 2017 ▸), binuclear complexes (Santoni *et al.*, 2013 ▸; Schmittel *et al.*,2006 ▸; Maekawa *et al.*, 2004 ▸), tetra­nuclear complexes (Schmittel *et al.*, 2005 ▸), one-dimensional coordination polymers (Koo *et al.*, 2003 ▸), two-dimensional coordination polymers (Bulut *et al.*, 2015 ▸; Jones *et al.* (2010 ▸), and numerous metallo-supra­molecular polymers (without reported crystal structures), see for example: Vaduvescu & Potvin, 2004 ▸; Nishimori *et al.*, 2007 ▸; Han *et al.*, 2008 ▸; Schwarz *et al.*, 2010 ▸; Ding *et al.*, 2012 ▸; Muronoi *et al.*, 2013 ▸; Szczerba *et al.*, 2014 ▸; Munzert *et al.*, 2016 ▸; Meded *et al.*, 2017 ▸; Bera *et al.*, 2018 ▸.
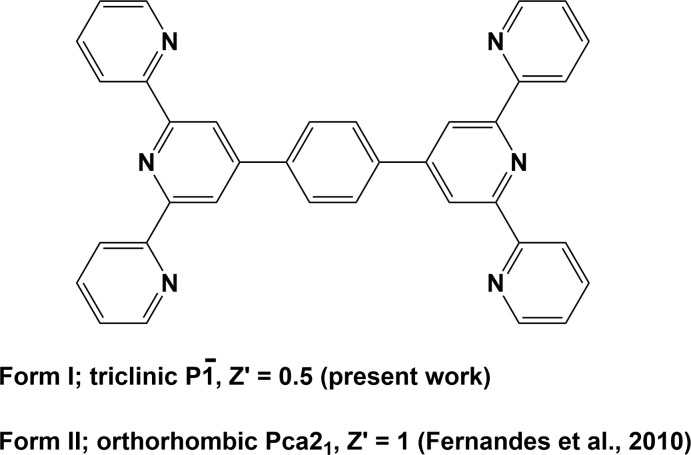


A search of the Cambridge Structural Database (CSD, Version 5.40, update August 2019; Groom *et al.*, 2016 ▸) for the title compound yielded only nine hits (see supporting information), which included the report on the structure of the ortho­rhom­bic polymorph, Form II, by Fernandes *et al.* (2010 ▸).

## Structural commentary   

**Figure 1:**
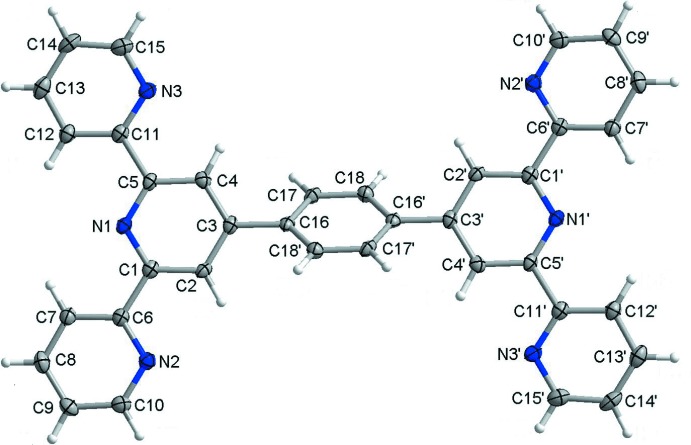
The mol­ecular structure of the title triclinic polymorph (Form I), with atom labelling [symmetry code (’): −*x*, −*y*, −*z*]. Displacement ellipsoids are drawn at the 50% probability level.

The mol­ecular structure of the title triclinic polymorph (Form I) is illustrated in Fig. 1 ▸. The mol­ecule is located about a crystallographic centre of symmetry in the middle of the central benzene ring (C16–C18/C16′–C18′), hence the mol­ecule has a higher symmetry (point group *C_i_*) than that observed for the ortho­rhom­bic polymorph, Form II (Fernandes *et al.*, 2010 ▸), which has point group *C*_1_. In Form I the side pyridine rings (N2/C6–C10 and N3/C11–C15) are rotated slightly with respect to the central pyridine ring (N1/C1–C5), with dihedral angles of 8.91 (8) and 10.41 (8)°, respectively. Opposite central pyridine rings (N1/C1–C5 and N1′/C1′–C5′) are coplanar by symmetry, and the angle between them and the central benzene ring (C16–C18/C16′–C18′) is 49.98 (8)° [symmetry code: (’) −*x*, −*y*, −*z*]. The nitro­gen atoms of the pyridine rings inside the 2,2′:6′,2′′-terpyridine (terpy) entities, N3⋯N1⋯N2, lie in *trans–trans* positions.

**Figure 2:**
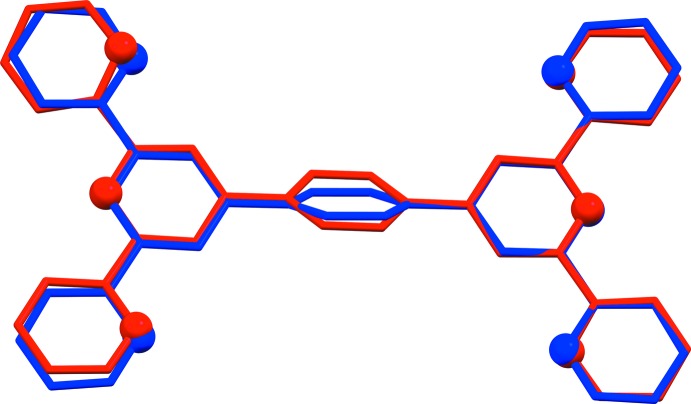
A structural overlay of the title triclinic polymorph (Form I; blue) and the ortho­rhom­bic polymorph (Form II; red), drawn using *Mercury* (Macrae *et al.*, 2008 ▸).

In the ortho­rhom­bic polymorph, Form II, all the angles between side and central pyridine rings of the terpy units are different (because of the lack of symmetry elements inside the mol­ecule), *viz*. 24.86 (12) and 5.10 (12)° on one side and 6.30 (11) and 8.21 (12)° on the opposite side. The dihedral angles between the central pyridine rings of the terpy units and the central benzene ring are 34.95 (11) and 36.17 (11)°. A structural overlay of the mol­ecules of the two polymorphs (r.m.s. deviation = 0.0705 Å), illustrating the differences in their conformation, is given in Fig. 2 ▸ (*Mercury*; Macrae *et al.*, 2008 ▸).

## Supra­molecular features   

**Figure 3:**
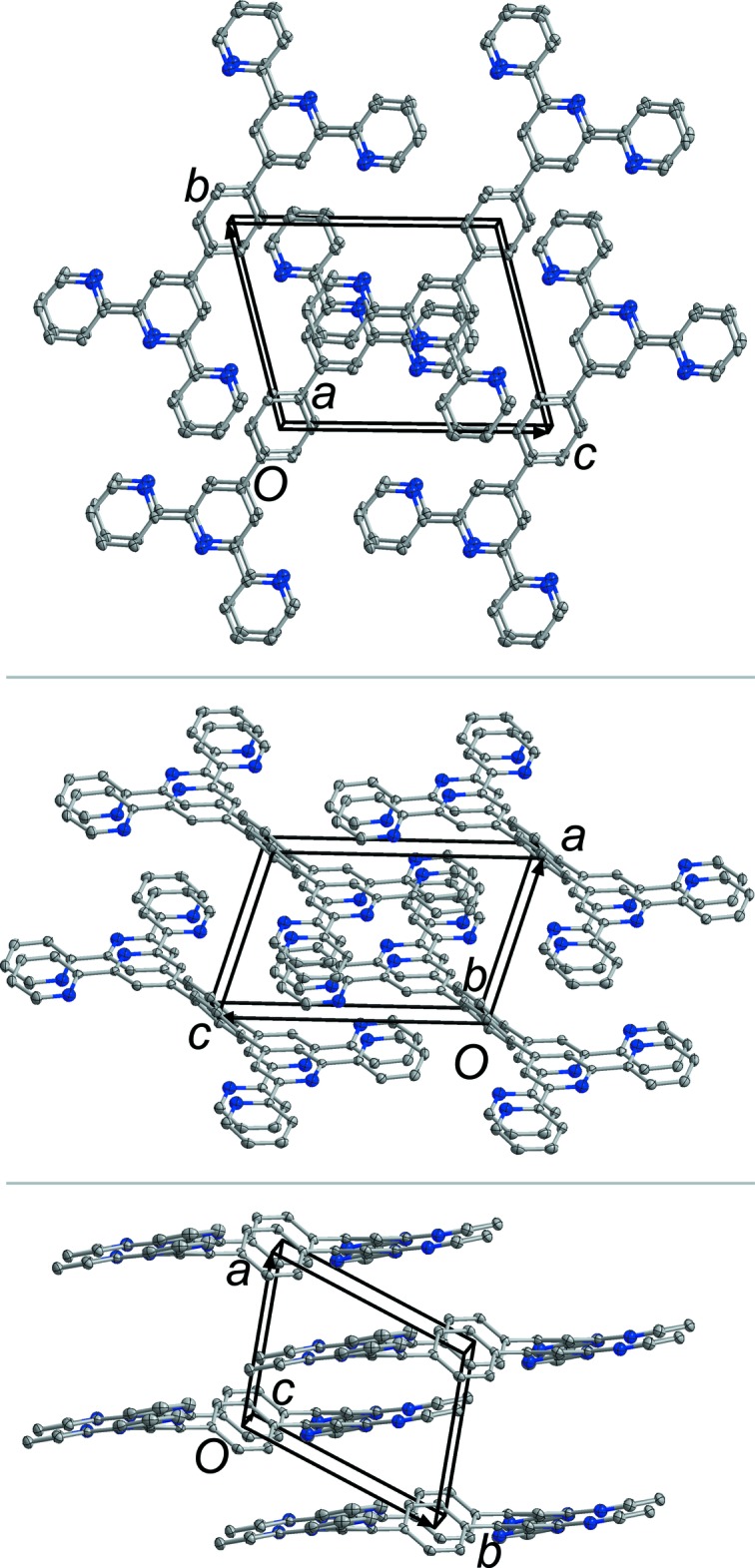
The crystal packing of the title triclinic polymorph (Form I) viewed along the *a* (top), *b* (middle) and *c* (bottom) axes.

In the crystal of the title polymorph, Form I, the mol­ecules stack along the *a*-, *b*- and *c*-axis directions (Fig. 3 ▸). They are linked by C—H⋯π inter­actions (Table 1 ▸) and offset π–π inter­actions, which are summarized in Table 2 ▸ for both Form I and Form II. It is inter­esting to note that the centroid–centroid distances and the offset distances are significantly shorter for Form II. An additional difference between the two polymorphs is the character of stacking: in Form II mol­ecules form several two-dimensional stacks, which are perpendicular to each another, while in Form I the stacking is three-dimensional.

## Hirshfeld surfaces and two-dimensional fingerprint plots   

The Hirshfeld surface analysis (Spackman & Jayatilaka, 2009 ▸) and the associated two-dimensional fingerprint plots (McKinnon *et al.*, 2007 ▸) were performed with *CrystalExplorer17* (Turner *et al.*, 2017 ▸). For an excellent explanation of the use of Hirshfeld surface analysis and other calculations to study mol­ecular packing, see the recent article by Tiekink and collaborators (Tan *et al.*, 2019 ▸).

The Hirshfeld surfaces are colour-mapped with the normalized contact distance, *d*_norm_, from red (distances shorter than the sum of the van der Waals radii) through white to blue (distances longer than the sum of the van der Waals radii).

**Figure 4:**
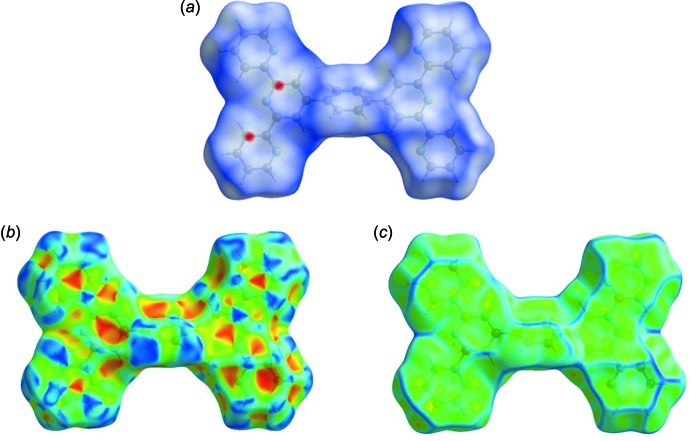
(*a*) The Hirshfeld surface of Form I, mapped over *d*_norm_, plotted in the range −0.0541 to 1.3209 a.u., (*b*) the Hirshfeld surface of Form I, mapped over the shape-index and (*c*) the Hirshfeld surface of Form I, mapped over the curvedness.

The Hirshfeld surface of Forms I and II, mapped over *d*_norm_ are given in Fig. 4 ▸ *a* and 5 ▸ *a*, respectively, where short inter­atomic contacts are indicated by the faint red spots. The π–π stacking is confirmed by the small blue regions surrounding bright-red spots in the various aromatic rings (Fig. 4 ▸ *b* and 5*b*) on the Hirshfeld surface mapped over the shape-index, and by the flat regions around the aromatic regions in Fig. 4 ▸ *c* and 5*c*, the Hirshfeld surface mapped over the curvedness.

**Figure 6:**
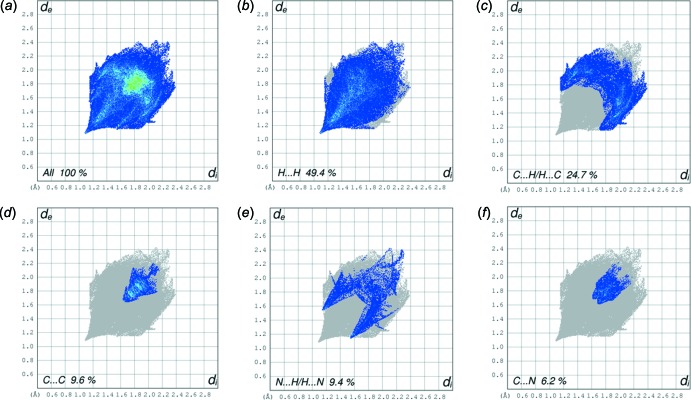
The full two-dimensional fingerprint plot for Form I, and fingerprint plots delineated into H⋯H, C⋯H/H⋯C, C⋯C, N⋯H/H⋯N and C⋯N contacts.

The fingerprint plots for Forms I and II, are given in Figs. 6 ▸ and 7 ▸. They reveal that the principal inter­molecular contacts in the crystal of Form I are H⋯H at 49.4% (Fig. 6 ▸ *b*), C⋯H/H⋯C at 24.7% (Fig. 6 ▸ *c*), C⋯C at 9.6% (Fig. 6 ▸ *d*), N⋯H/H⋯N at 9.4% (Fig. 6 ▸ *e*) and C⋯N at 6.2% (Fig. 6 ▸ *f*).

**Figure 7:**
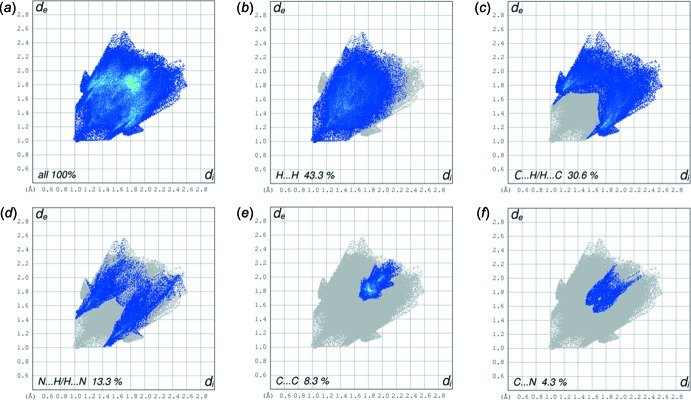
The full two-dimensional fingerprint plot for Form II, and fingerprint plots delineated into H⋯H, C⋯H/H⋯C, N⋯H/H⋯N, C⋯C and C⋯N contacts.

The principal inter­molecular contacts in the crystal of Form II are H⋯H at 43.3% (Fig. 7 ▸ *b*), C⋯H/H⋯C at 30.6% (Fig. 7 ▸ *c*), N⋯H/H⋯N at 13.3% (Fig. 7 ▸ *d*), C⋯C at 8.3% (Fig. 7 ▸ *e*) and C⋯N at 4.3% (Fig. 7 ▸ *f*). Here, the C⋯H/H⋯C and N⋯H/H⋯N contacts at 30.6 and 13.3%, respectively, are more important than those in Form I at 24.7 and 9.4%, respectively.

## Synthesis and crystallization   

1,4-Bis([2,2′:6′,2′′-terpyridin]-4′-yl)benzene was synthesized according to the literature procedure (Winter *et al.*, 2006 ▸). YCl_3_ (99.9%, Strem) was purchased and used as received. Solvents (DMF, toluene) were dried using standard techniques and stored with mol­ecular sieves in flasks with a J. Young valve.

YCl_3_ (2 mg, 0.01 mmol), 1,4-bis­([2,2′:6′,2′′-terpyridin]-4′-yl)benzene (0.5 mg, 0.001 mmol) and 1 ml DMF were filled together under inert conditions in a self-made Duran^(R)^ glass ampoule (outer ø 10 mm, wall thickness 1 mm). The ampoule was sealed under vacuum and placed in a resistance heating oven with a thermal control (Eurotherm 2416). The heating program was as follows: heating up to 503 K in 30 min, holding temperature for 8 h, cooling down to RT uncontrollably. The ampoule was then taken out of the oven and a star-like net of needle-shaped single crystals was observed. The ampoule was heated again as previously but up to 523 K and then cooled down to RT uncontrollably. Now only a few plate-shaped single crystals were present. The ampoule was unsealed, the solution removed and the remaining single crystals were washed with toluene (1 ml).

## Refinement   

Crystal data, data collection and structure refinement details are summarized in Table 3 ▸. The H atoms were included in calculated positions and refined as riding on the parent C atom: C—H = 0.95 Å with *U*_iso_(H) = 1.2*U*_eq_(C).

## Supplementary Material

Crystal structure: contains datablock(s) I, global. DOI: 10.1107/S2056989019015810/su5525sup1.cif

Structure factors: contains datablock(s) I. DOI: 10.1107/S2056989019015810/su5525Isup2.hkl

CSD search. DOI: 10.1107/S2056989019015810/su5525sup3.pdf

Supporting information file. DOI: 10.1107/S2056989019015810/su5525Isup4.cml

CCDC reference: 1967605

Additional supporting information:  crystallographic information; 3D view; checkCIF report

## Figures and Tables

**Figure 5:**
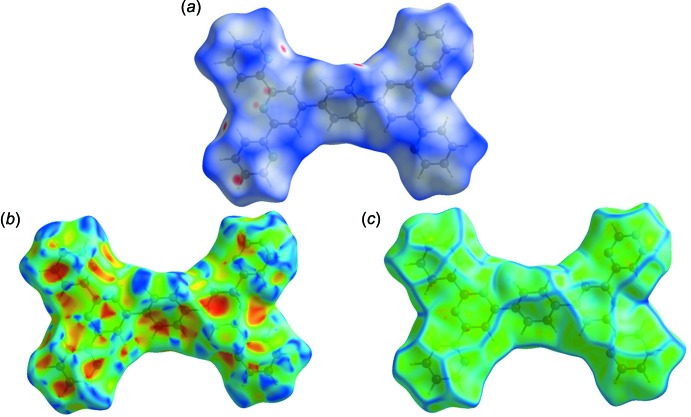
(*a*) The Hirshfeld surface of Form II, mapped over *d*_norm_, plotted in the range −0.1446 to 1.2077 a.u., (*b*) the Hirshfeld surface of Form II, mapped over the shape-index and (*c*) the Hirshfeld surface of Form II, mapped over the curvedness.

**Table 1:** Hydrogen-bond geometry (Å, °) *Cg*2 is the centroid of the N2/C6–C10 ring.

*D*—H⋯*A*	*D*—H	H⋯*A*	*D*⋯*A*	*D*—H⋯*A*
C17—H17⋯*Cg*2^i^	0.96	2.99	3.682 (2)	131

Symmetry code: (i) 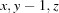.

**Table 2:** π–π stacking inter­actions (Å, °) for Form I and Form II Form I: *Cg*1, *Cg*2 and *Cg*3 are the centroids of the N1/C1–C5, N2/C6–C10 and N3/C11–C15 rings, respectively. Form II: *Cg*1 and *Cg*2 are the centroids of the N1/C1–C5 and N2/C6–C10 rings, respectively (Fernandes *et al.*, 2010 ▸).

*CgI*	*CgJ*	*CgI*⋯*CgJ*	α	β	γ	*CgI*_Perp	*CgJ*_Perp	offset
Form I								
*Cg*1	*Cg*3^ii^	3.6421 (16)	8.91 (8)	18.6	17.9	3.4648 (6)	3.4525 (8)	1.160
*Cg*2	*Cg*3^iii^	3.7813 (16)	4.43 (8)	26.0	24.8	3.4312 (7)	3.3990 (8)	1.657
								
Form II								
*Cg*1	*Cg*2^iv^	3.5138 (15)	4.20 (12)	10.9	14.9	3.3963 (12)	3.4501 (9)	0.666
*Cg*2	*Cg*1^v^	3.5140 (15)	4.20 (12)	14.9	10.9	3.4503 (9)	3.3963 (12)	0.902

Symmetry codes (ii) −*x*, −*y* + 1, −*z* + 1; (iii) −*x* + 1, −*y* + 1, −*z* + 1; (iv) *x* − 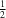, −*y*, *z*; (v) *x* + 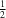, −*y*, *z*.

**Table 3:** Experimental details

Crystal data	
Chemical formula	C_36_H_24_N_6_
*M*_r_	540.61
Crystal system, space group	Triclinic, *P* 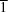
Temperature (K)	100
*a*, *b*, *c* (Å)	7.312 (2), 8.847 (3), 11.039 (3)
α, β, γ (°)	100.050 (7), 102.247 (6), 104.314 (7)
*V* (Å^3^)	656.4 (3)
*Z*	1
Radiation type	Mo *K*α
μ (mm^−1^)	0.08
Crystal size (mm)	0.53 × 0.30 × 0.23

Data collection	
Diffractometer	Bruker X8 APEXII
Absorption correction	Multi-scan (*SADABS*; Bruker, 2017 ▸)
*T*_min_, *T*_max_	0.764, 0.958
No. of measured, independent and observed [*I* > 2σ(*I*)] reflections	10437, 2918, 1953
*R*_int_	0.049
(sin θ/λ)_max_ (Å^−1^)	0.643

Refinement	
*R*[*F* ^2^ > 2σ(*F* ^2^)], *wR*(*F* ^2^), *S*	0.047, 0.133, 1.09
No. of reflections	2918
No. of parameters	190
H-atom treatment	H-atom parameters constrained
Δρ_max_, Δρ_min_ (e Å^−3^)	0.27, −0.22

Computer programs: *APEX3* and *SAINT* (Bruker, 2017 ▸), *SHELXT* (Sheldrick, 2015*a* ▸), *SHELXL* (Sheldrick, 2015*b* ▸), *shelXle* (Hübschle *et al.*, 2011 ▸), *Mercury* (Macrae *et al.*, 2008 ▸), *PLATON* (Spek, 2009 ▸) and *publCIF* (Westrip, 2010 ▸).

## supplementary crystallographic information

### Crystal data

**Table:**

C_36_H_24_N_6_	*Z* = 1
*M**_r_* = 540.61	*F*(000) = 282
Triclinic, *P*1	*D*_x_ = 1.368 Mg m^−^^3^
Hall symbol: -P 1	Mo *K*α radiation, λ = 0.71073 Å
*a* = 7.312 (2) Å	Cell parameters from 3259 reflections
*b* = 8.847 (3) Å	θ = 2.5–27.1°
*c* = 11.039 (3) Å	µ = 0.08 mm^−^^1^
α = 100.050 (7)°	*T* = 100 K
β = 102.247 (6)°	Plate, colourless
γ = 104.314 (7)°	0.53 × 0.30 × 0.23 mm
*V* = 656.4 (3) Å^3^	

### Data collection

**Table:**

Bruker X8 APEXII diffractometer	2918 independent reflections
Radiation source: rotating-anode (Nonius FR-591)	1953 reflections with *I* > 2σ(*I*)
Multi-layer mirror monochromator	*R*_int_ = 0.049
Detector resolution: 8.333 pixels mm^-1^	θ_max_ = 27.2°, θ_min_ = 1.9°
φ and ω scans	*h* = −9→5
Absorption correction: multi-scan (SADABS; Bruker, 2017)	*k* = −11→11
*T*_min_ = 0.764, *T*_max_ = 0.958	*l* = −14→14
10437 measured reflections	

### Refinement

**Table:**

Refinement on *F*^2^	Primary atom site location: dual
Least-squares matrix: full	Secondary atom site location: difference Fourier map
*R*[*F*^2^ > 2σ(*F*^2^)] = 0.047	Hydrogen site location: inferred from neighbouring sites
*wR*(*F*^2^) = 0.133	H-atom parameters constrained
*S* = 1.09	*w* = 1/[\s^2^(*F*_o_^2^) + (0.0552*P*)^2^ + 0.1381*P*] where *P* = (*F*_o_^2^ + 2*F*_c_^2^)/3
2918 reflections	(Δ/σ)_max_ < 0.001
190 parameters	Δρ_max_ = 0.27 e Å^−^^3^
0 restraints	Δρ_min_ = −0.22 e Å^−^^3^

### Special details

**Table:**

Geometry. All esds (except the esd in the dihedral angle between two l.s. planes) are estimated using the full covariance matrix. The cell esds are taken into account individually in the estimation of esds in distances, angles and torsion angles; correlations between esds in cell parameters are only used when they are defined by crystal symmetry. An approximate (isotropic) treatment of cell esds is used for estimating esds involving l.s. planes.

### Fractional atomic coordinates and isotropic or equivalent isotropic displacement parameters (Å^2^)

**Table:**

	*x*	*y*	*z*	*U*_iso_*/*U*_eq_	
N1	0.31314 (19)	0.57168 (16)	0.39738 (13)	0.0184 (3)	
N2	0.4458 (2)	0.73483 (17)	0.13929 (13)	0.0213 (3)	
N3	0.0878 (2)	0.29403 (17)	0.56336 (13)	0.0227 (4)	
C1	0.3286 (2)	0.5741 (2)	0.27836 (16)	0.0180 (4)	
C2	0.2523 (2)	0.4377 (2)	0.17767 (16)	0.0183 (4)	
H2	0.260141	0.444780	0.093931	0.022*	
C3	0.1647 (2)	0.2912 (2)	0.20071 (15)	0.0178 (4)	
C4	0.1538 (2)	0.2879 (2)	0.32419 (16)	0.0190 (4)	
H4	0.098885	0.189236	0.344145	0.023*	
C5	0.2242 (2)	0.4309 (2)	0.41872 (15)	0.0170 (4)	
C6	0.4396 (2)	0.7297 (2)	0.25945 (15)	0.0177 (4)	
C7	0.5401 (2)	0.8601 (2)	0.36334 (16)	0.0223 (4)	
H7	0.531212	0.853493	0.446984	0.027*	
C8	0.6527 (2)	0.9991 (2)	0.34305 (18)	0.0261 (4)	
H8	0.722880	1.089152	0.412386	0.031*	
C9	0.6610 (2)	1.0042 (2)	0.22011 (17)	0.0245 (4)	
H9	0.737656	1.097586	0.203028	0.029*	
C10	0.5551 (2)	0.8702 (2)	0.12213 (17)	0.0236 (4)	
H10	0.560586	0.875011	0.037631	0.028*	
C11	0.2008 (2)	0.4309 (2)	0.54952 (15)	0.0187 (4)	
C12	0.2926 (2)	0.5660 (2)	0.65033 (16)	0.0220 (4)	
H12	0.370162	0.661960	0.637260	0.026*	
C13	0.2681 (3)	0.5569 (2)	0.77001 (17)	0.0267 (4)	
H13	0.330137	0.646686	0.840845	0.032*	
C14	0.1533 (3)	0.4169 (2)	0.78556 (17)	0.0270 (4)	
H14	0.134352	0.408052	0.866839	0.032*	
C15	0.0662 (3)	0.2893 (2)	0.67977 (17)	0.0277 (4)	
H15	−0.013662	0.192846	0.690615	0.033*	
C16	0.0823 (2)	0.14143 (19)	0.09699 (15)	0.0178 (4)	
C17	0.1252 (2)	0.0007 (2)	0.11349 (16)	0.0197 (4)	
H17	0.210899	0.000508	0.191289	0.024*	
C18	−0.0443 (2)	0.1397 (2)	−0.01748 (15)	0.0200 (4)	
H18	−0.075227	0.234999	−0.029841	0.024*	

### Atomic displacement parameters (Å^2^)

**Table:**

	*U*^11^	*U*^22^	*U*^33^	*U*^12^	*U*^13^	*U*^23^
N1	0.0168 (7)	0.0215 (8)	0.0160 (8)	0.0058 (6)	0.0032 (6)	0.0033 (6)
N2	0.0215 (7)	0.0226 (8)	0.0191 (8)	0.0060 (6)	0.0045 (6)	0.0053 (6)
N3	0.0234 (8)	0.0268 (8)	0.0182 (8)	0.0065 (6)	0.0064 (6)	0.0063 (6)
C1	0.0136 (8)	0.0223 (9)	0.0174 (9)	0.0066 (7)	0.0024 (7)	0.0035 (7)
C2	0.0162 (8)	0.0247 (9)	0.0129 (9)	0.0049 (7)	0.0033 (7)	0.0036 (7)
C3	0.0136 (8)	0.0216 (9)	0.0162 (9)	0.0054 (7)	0.0021 (7)	0.0013 (7)
C4	0.0158 (8)	0.0198 (9)	0.0201 (9)	0.0039 (7)	0.0039 (7)	0.0044 (7)
C5	0.0129 (8)	0.0217 (9)	0.0144 (9)	0.0048 (7)	0.0017 (6)	0.0018 (7)
C6	0.0140 (8)	0.0208 (9)	0.0179 (9)	0.0062 (7)	0.0034 (7)	0.0034 (7)
C7	0.0216 (9)	0.0241 (9)	0.0187 (9)	0.0049 (7)	0.0047 (7)	0.0017 (7)
C8	0.0213 (9)	0.0216 (10)	0.0287 (11)	0.0023 (7)	0.0029 (8)	−0.0002 (8)
C9	0.0191 (8)	0.0220 (9)	0.0308 (11)	0.0029 (7)	0.0066 (8)	0.0072 (8)
C10	0.0229 (9)	0.0269 (10)	0.0223 (10)	0.0071 (8)	0.0066 (7)	0.0089 (8)
C11	0.0156 (8)	0.0234 (9)	0.0171 (9)	0.0081 (7)	0.0021 (7)	0.0040 (7)
C12	0.0205 (9)	0.0250 (10)	0.0193 (9)	0.0080 (7)	0.0034 (7)	0.0031 (8)
C13	0.0263 (9)	0.0344 (11)	0.0188 (10)	0.0144 (8)	0.0022 (8)	0.0018 (8)
C14	0.0277 (9)	0.0431 (12)	0.0157 (9)	0.0169 (9)	0.0081 (8)	0.0089 (8)
C15	0.0285 (10)	0.0344 (11)	0.0238 (10)	0.0096 (8)	0.0097 (8)	0.0123 (9)
C16	0.0148 (8)	0.0207 (9)	0.0154 (9)	0.0011 (7)	0.0054 (7)	0.0019 (7)
C17	0.0172 (8)	0.0264 (9)	0.0132 (9)	0.0052 (7)	0.0020 (6)	0.0030 (7)
C18	0.0201 (8)	0.0200 (9)	0.0191 (9)	0.0052 (7)	0.0051 (7)	0.0040 (7)

### Geometric parameters (Å, º)

**Table:**

N1—C5	1.340 (2)	C8—C9	1.379 (3)
N1—C1	1.346 (2)	C8—H8	0.9500
N2—C10	1.334 (2)	C9—C10	1.385 (2)
N2—C6	1.345 (2)	C9—H9	0.9500
N3—C15	1.334 (2)	C10—H10	0.9500
N3—C11	1.340 (2)	C11—C12	1.393 (2)
C1—C2	1.393 (2)	C12—C13	1.384 (2)
C1—C6	1.489 (2)	C12—H12	0.9500
C2—C3	1.389 (2)	C13—C14	1.374 (3)
C2—H2	0.9500	C13—H13	0.9500
C3—C4	1.387 (2)	C14—C15	1.383 (3)
C3—C16	1.487 (2)	C14—H14	0.9500
C4—C5	1.395 (2)	C15—H15	0.9500
C4—H4	0.9500	C16—C17	1.389 (2)
C5—C11	1.490 (2)	C16—C18	1.394 (2)
C6—C7	1.396 (2)	C17—C18^i^	1.388 (2)
C7—C8	1.383 (2)	C17—H17	0.9500
C7—H7	0.9500	C18—H18	0.9500
			
C5—N1—C1	117.93 (14)	C10—C9—H9	120.7
C10—N2—C6	117.38 (14)	N2—C10—C9	123.93 (17)
C15—N3—C11	117.47 (15)	N2—C10—H10	118.0
N1—C1—C2	122.49 (15)	C9—C10—H10	118.0
N1—C1—C6	116.71 (14)	N3—C11—C12	122.76 (16)
C2—C1—C6	120.75 (15)	N3—C11—C5	115.99 (14)
C3—C2—C1	119.43 (15)	C12—C11—C5	121.25 (15)
C3—C2—H2	120.3	C13—C12—C11	118.34 (17)
C1—C2—H2	120.3	C13—C12—H12	120.8
C4—C3—C2	118.00 (15)	C11—C12—H12	120.8
C4—C3—C16	120.29 (15)	C14—C13—C12	119.44 (17)
C2—C3—C16	121.70 (15)	C14—C13—H13	120.3
C3—C4—C5	119.28 (16)	C12—C13—H13	120.3
C3—C4—H4	120.4	C13—C14—C15	118.26 (17)
C5—C4—H4	120.4	C13—C14—H14	120.9
N1—C5—C4	122.72 (15)	C15—C14—H14	120.9
N1—C5—C11	117.46 (14)	N3—C15—C14	123.72 (18)
C4—C5—C11	119.82 (15)	N3—C15—H15	118.1
N2—C6—C7	122.30 (16)	C14—C15—H15	118.1
N2—C6—C1	116.79 (14)	C17—C16—C18	119.01 (15)
C7—C6—C1	120.83 (15)	C17—C16—C3	120.93 (15)
C8—C7—C6	119.20 (16)	C18—C16—C3	120.04 (15)
C8—C7—H7	120.4	C18^i^—C17—C16	120.68 (16)
C6—C7—H7	120.4	C18^i^—C17—H17	119.7
C9—C8—C7	118.65 (16)	C16—C17—H17	119.7
C9—C8—H8	120.7	C17^i^—C18—C16	120.31 (16)
C7—C8—H8	120.7	C17^i^—C18—H18	119.8
C8—C9—C10	118.53 (16)	C16—C18—H18	119.8
C8—C9—H9	120.7		

Symmetry code: (i) −*x*, −*y*, −*z*.

### Hydrogen-bond geometry (Å, º)

*Cg*2 is the centroid of the N2/C6–C10 ring.

**Table:**

*D*—H···*A*	*D*—H	H···*A*	*D*···*A*	*D*—H···*A*
C17—H17···*Cg*2^ii^	0.96	2.99	3.682 (2)	131

Symmetry code: (ii) *x*, *y*−1, *z*.
