# Supplementary material for: The crystal structure of the triclinic polymorph of 1,4-bis­([2,2′:6′,2′′-terpyridin]-4′-yl)benzene
Source: Acta Crystallogr E Crystallogr Commun. 2019 Nov 29;75(Pt 12):1947–51. doi: 10.1107/S2056989019015810 (PMC6895940; doi:10.1107/S2056989019015810)

Search Overview

Search:	search1
Date/Time done:	Wed Oct 30 10:13:58 2019
Database(s):	CSD version 5.40 updates (Feb 2019)<br>CSD version 5.40 (November 2018)<br>CSD version 5.40 updates (May 2019)<br>CSD version 5.40 updates (Aug 2019)
Restriction Info:	No refcode restrictions applied
Filters:	None
Percentage Completed:	100%
Number of Hits:	9

Single query used. Search found structures that:

match

Query 1

Query 1

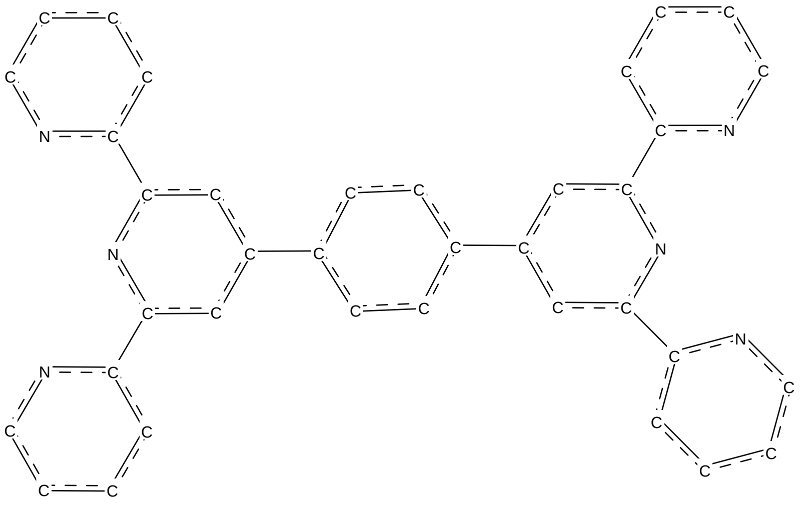

Search: search1 (Wed Oct 30 10:13:58 2019): Hits 1-4

HURGUY

Reference: A.Bulut, Y.Zorlu, E.Kirpi, A.Cetinkaya, M.Worle, J.Beckmann, G.Yucesan (2015) *Cryst.Growth Des.* ,**15,**5665

Formula: $(C_{61} H_{44} Cu_2 N_6 O_{12} P_4)n$

Compound Name: catena-[(μ-(4-(bis(4-(hydroxyphosphinato)phenyl)(4-phosphonophenyl) methyl)phenyl)phosphonato)-(μ-4',4''-(1,4-phenylene)bis(2,2':6',2''-terpyridine))-di-copper unknown solvate]

Space Group:	P-1	***Cell:***	***a*** 11.895(0)	***b*** 13.128(1)	***c*** 22.391(1)		
Space Group No.:	2	***(Å,°)***	α 77.04(0)	β 89.77(0)	γ 69.06(0)		
R-Factor (%):	5.10	***Temperature(K):***	299	***Density(g/cm^3):***	1.366		

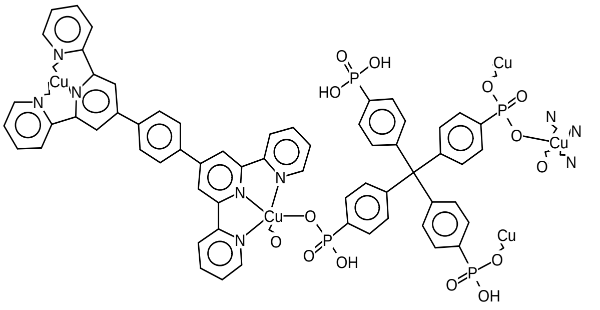

LIGYEH

Reference: M.-P.Santoni, F.Nastasi, S.Campagna, G.S.Hanan, B.Hasenknopf, I.Ciofini (2013) *Dalton Trans.* ,**42,**5281

Formula: $C_{55} H_{36} Br_1 N_{11} Ru_1{}^{2+},2(F_6 P_1{}^{1-})$

Compound Name: (2-(4-Bromophenyl)-4,6-bis(pyridin-2-yl)-1,3,5-triazine)-(4'-(4-(2,2':6',2''-terpyridin-4'-yl)phenyl)-2,2':6',2''-terpyridine)-ruthenium(ii) bis(hexafluorophosphate)

Space Group:	P-1	***Cell:***	***a*** 9.259(1)	***b*** 14.846(3)	***c*** 21.138(4)
Space Group No.:	2	***(Å,°)***	α 108.12(0)	β 98.11(0)	γ 93.99(0)
R-Factor (%):	16.62	***Temperature(K):***	100	***Density(g/cm^3):***	1.618

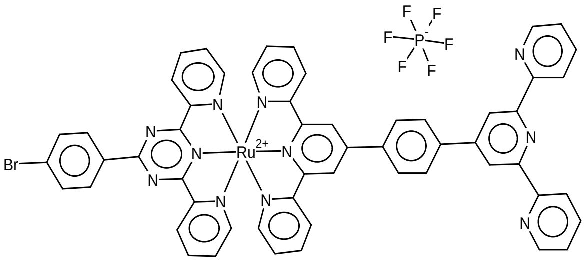

NEFCIN

Reference: B.Laramee-Milette, G.S.Hanan (2017) *Chem.Commun.* , **53,**10496

Formula: $C_{55} H_{43} N_{11} Ru_1{}^{2+},2(F_6 P_1{}^{1-})$

Compound Name: {2^4-[4-([1^2,2^2:2^6,3^2-terpyridin]-2^4-yl)phenyl]-1^2,2^2:2^6,3^2-terpyridine}-[2-(3,4,7,8-tetrahydro-2H-pyrimido[1,2-a]pyrimidin-1(6H)-yl)-1,10-phenanthroline]-ruthenium bis(hexafluorophosphate) unknown solvate

Space Group:	P-1	***Cell:***	***a*** 9.335(1)	***b*** 12.808(3)	***c*** 23.398(5)
Space Group No.:	2	***(Å,°)***	α 97.10(0)	β 100.18(0)	γ 92.63(0)
R-Factor (%):	14.70	***Temperature(K):***	150	***Density(g/cm^3):***	1.522

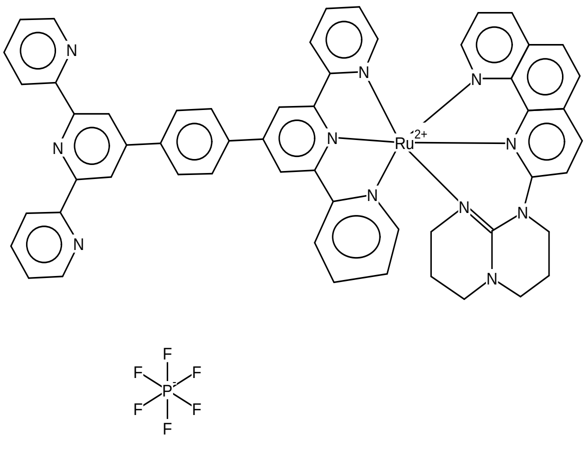

RUTPIG

Reference: S.Jones, Hongxue Liu, W.Ouellette, K.Schmidtke, C.J.O'Connor, J.Zubieta (2010) *Inorg.Chem.Commun.* ,**13,**491

Formula: $(C_{80} H_{70} Cu_4 F_2 Mo_8 N_{12} O_{38} P_4)n,4n(H_2 O_1)$

Compound Name: catena-((μ_8-1,4-Butylenebis(phosphonato))-(μ_4-1,4-butylenebis(hydrogen phosphonato))-bis(μ_4-fluoro)-bis(μ_2-1,4-bis(2, 2':6',2''-terpyridin-4'-yl)benzene)-decakis(μ_2-oxo)-diaqua-tetradecaoxo-tetra-copper-octa-molybdenum tetrahydrate)

Space Group:	P-1	***Cell:***	***a*** 11.660(0)	***b*** 12.825(0)	***c*** 17.220(1)
Space Group No.:	2	***(Å,°)***	α 96.32(0)	β 104.68(0)	γ 106.91(0)
R-Factor (%):	3.55	***Temperature(K):***	90	***Density(g/cm^3):***	2.178

H_2O

n-$Mo_8Cu_4[\mu_8\text{-}C_4H_8O_6P_2]$--
--$[\mu_4\text{-}C_4H_{10}O_6P_2\text{-}\kappa^4O,O',O'',O''']$$[\mu_4\text{-}F]_2$--
--$[\mu_2\text{-}C_{36}H_{24}N_6\text{-}\kappa^6N,N',N'',N''',N'''',N''''']_2[\mu_2\text{-}O]_{10}[H_2O]_2[O]_{14}$

Search: search1 (Wed Oct 30 10:13:58 2019): Hits 5-8

TAVGED

Reference: M.Schmittel, V.Kalsani, R.S.K.Kishore, H.Colfen, J.W.Bats (2005) *J.Am.Chem.Soc.* ,**127,**11544

Formula: $C_{208} H_{156} N_{20} O_4 Zn_4{}^{8+}$,8($C_1 F_3 O_3 S_1{}^{1-}$),2($C_1 H_2 Cl_2$),$C_1 H_4 O_1$

Compound Name: bis(μ2-1,4-bis(2-(2-(4-hydroxy-2,6-dimethylphenyl)-9-mesityl-1,10-phenanthrolin-3-yl)ethynyl)benzene-N,N',N'',N''')-bis(μ2-benzene-1,4-bis(2,2':6',2''-terpyridin-4'-yl))-tetra-zinc(ii) octakis(trifluoromethanesulfonate) dichloromethane methanol solvate

Space Group:	P21/n	***Cell:***	***a***	21.715(6)	***b***	19.196(6)	***c***	28.620(7)
Space Group No.:	14	***(Å,°)***	α	90.00	β	94.85(1)	γ	90.00

R-Factor (%): 23.15 ***Temperature(K):*** 157 ***Density(g/cm³):*** 1.301

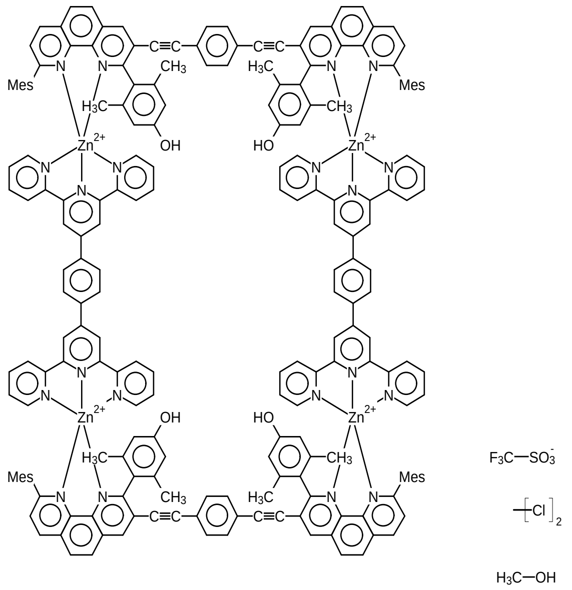

UJUFAG

Reference: Bon-Kweon Koo, L.Bewley, V.Golub, R.S.Rarig, E.Burkholder, C.J.O'Connor, J.Zubieta (2003) *Inorg.Chim.Acta* ,**351,** 167

Formula: ($C_{36} H_{24} Cu_2 N_6 O_8 S_2$)n,2n($H_2 O_1$)

Compound Name: catena-((μ2-1,4-bis(2,2':6',2''-Terpyridine-4'-yl)benzene)-bis(μ2-sulfato)-di-copper(ii) dihydrate)

Space Group:	P-1	***Cell:***	***a***	8.534(0)	***b***	10.093(0)	***c***	10.534(0)
Space Group No.:	2	***(Å,°)***	α	94.05(0)	β	103.13(0)	γ	113.89(0)

R-Factor (%): 2.51 ***Temperature(K):*** 293 ***Density(g/cm³):*** 1.873

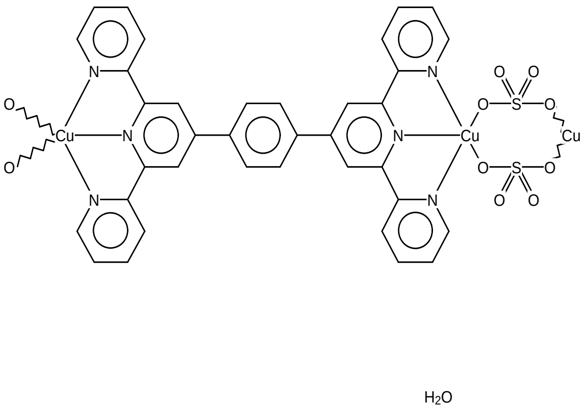

WADMEV

Reference: J.A.Fernandes, F.A.Almeida Paz, P.P.Lima, S.Alves Junior, L.D.Carlos (2010) *Acta Crystallogr.,Sect.E:Struct.Rep.Online* ,**66,**o3241

Formula: $C_{36} H_{24} N_6$

Compound Name: 4',4''-(1,4-Phenylene)bis(2,2':6',2''-terpyridine)

Synonym: 1,4-bis(2,2':6',2''-terpyridin-4'-yl)benzene

Space Group:	Pca21	***Cell:***	***a***	9.849(0)	***b***	10.063(0)	***c***	26.049(0)
Space Group No.:	29	***(Å,°)***	α	90.00	β	90.00	γ	90.00

R-Factor (%): 4.19 ***Temperature(K):*** 100 ***Density(g/cm³):*** 1.391

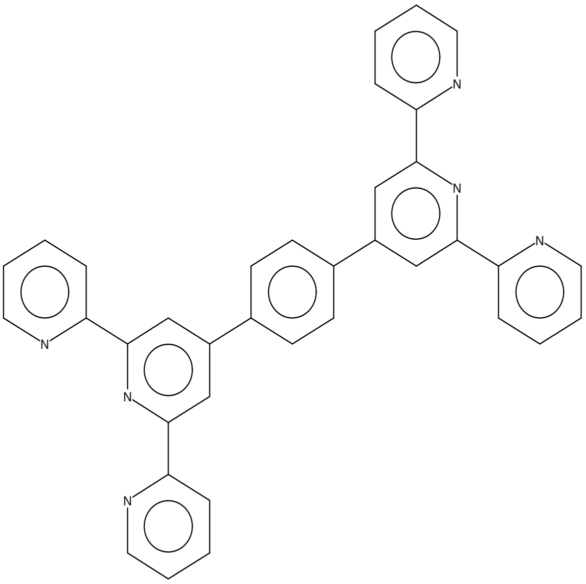

WEMLOQ

Reference: M.Schmitte, V.Kalsani, P.Mal, J.W.Bats (2006) *Inorg.Chem.* ,**45,**6370

Formula: $C_{96} H_{80} N_{10} Zn_2{}^{4+}$,4($C_1 F_3 O_3 S_1{}^{1-}$)

Compound Name: (μ2-1,4-bis(2,2':6',2''-terpyridin-4'-yl)phenylene)-bis((2,9-dimesityl-1,10-phenanthroline)-zinc(ii)) tetrakis(trifluoromethanesulfonate) unknown solvate

Space Group:	P-1	***Cell:***	***a***	11.521(2)	***b***	17.185(4)	***c***	18.164(4)
Space Group No.:	2	***(Å,°)***	α	114.94(1)	β	94.47(1)	γ	105.15(2)

R-Factor (%): 11.66 ***Temperature(K):*** 156 ***Density(g/cm³):*** 1.135

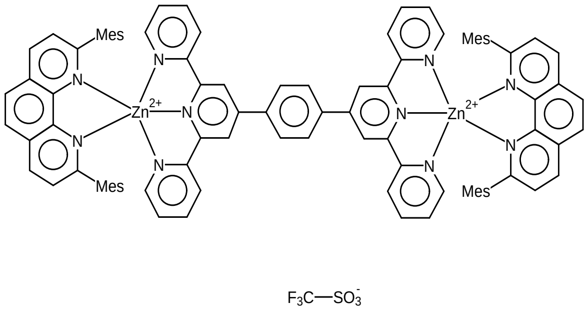

Search: search1 (Wed Oct 30 10:13:58 2019): Hit 9

XAHNEA

Reference:	M.Maekawa, T.Minematsu, H.Konaka, K.Sugimoto, T.Kuroda-Sowa, Y.Suenaga, M.Munakata (2004) *Inorg.Chim.Acta* ,**357,** 3456
Formula:	C_{108} H_{88} Ir_2 N_6 P_4 $^{2+}$,2(B_1 F_4 $^{1-}$),6(C_1 H_1 Cl_3)
Compound Name:	(μ_2-1,4-bis(2',6'-bis(2-pyridyl)-4'-pyridyl)benzene)-bis(cis-dihydro-trans-bis(triphenylphosphine)-iridium) bis(tetrafluoroborate) chloroform solvate
Synonym:	(μ_2-1,4-bis(2,2':6',2''-terpyridyl-4'-yl)benzene)-bis(cis-dihydro-trans-bis(triphenylphosphine)-iridium) bis(tetrafluoroborate) chloroform solvate

Space Group:	P-1	***Cell:***	***a*** 11.800(0)	***b*** 15.666(2)	***c*** 16.587(1)		
Space Group No.:	2	***(Å,°)***	α 93.44(0)	β 102.21(0)	γ 94.75(0)		
R-Factor (%):	3.49	***Temperature(K):***	150	***Density(g/cm³):***	1.600		

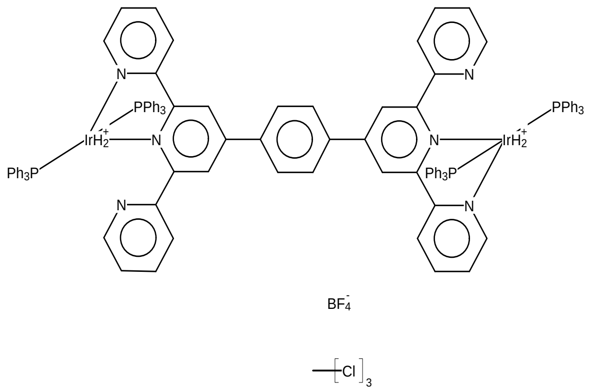

Supplement: Supplementary file 3 [file e-75-01947-sup3.pdf]
